# Action Mechanism of Fuzheng Fangai Pill Combined with Cyclophosphamide on Tumor Metastasis and Growth

**DOI:** 10.1155/2014/494528

**Published:** 2014-05-08

**Authors:** Sheng Liu, Xiao-min Wang, Guo-wang Yang

**Affiliations:** Oncology Department of Beijing Hospital of Traditional Chinese Medicine (TCM) affiliated to Capital Medical University, Beijing 100010, China

## Abstract

Fuzheng Fangai pill (FZFA), a traditional Chinese formula, is widely used for cancer treatment. Compared with other anticancer drugs, it is characterized by moderate and persistent efficacy with few side effects. The present paper emphasizes antitumor effect of FZFA combined with cyclophosphamide (CTX) on C57BL/6 mice subcutaneously injected with Lewis lung cancer cells, Comparing it with that of CTX. On the 21st day, a set of biochemical parameters were studied: the tumor weight and tumor volume, the inhibition rate of lung metastasis, the percentage and ratio of spleen CD4^+^IL-17^+^ Th17 (T helper cell 17, Th17 for short) and CD4^+^CD25^+^Foxp3^+^ Treg (T regulatory cell, Treg for short) cells, and the concentrations of IL-6, TGF-**β**, IL-17, IL-23, and IFN-**γ** in culture supernatants of CD4^+^ T lymphocytes were determined. The expression of the splenic Foxp3 and ROR**γ**t mRNA and JAK2, STAT3, and SOCS3 protein as also determined. The results show that compared with the model control group and CTX group, FZFA+CTX restored the ratio of spleen CD4^+^IL-17^+^ Th17 and CD4^+^CD25^+^ Foxp3^+^ Treg cells, and inhibited the inflammatory response including the nuclear SOCS3/JAK-STAT pathway, regulation of interleukins TGF-**β**, IL-6, IL-17, IL-23, and IFN-**γ**, and Foxp3 and ROR**γ**t gene expression in CD4^+^ T lymphocytes. We conclude that FZFA+CTX strongly reduced the growth and metastasis rate of Lewis lung cancer through inhibition of SOCS/JAK-STAT pathway and inflammatory cytokine responses. FZFA + CTX had greater activity than CTX alone.

## 1. Introduction

Tumor metastasis is closely related to the immune state of the human body [1]. During implementation of the National Natural Science Foundation of China project, we observed the effect of Fuzheng Fangai pill (FZFA) combined with cyclophosphamide (CTX) on the metastasis of several tumor types. Our results showed that FZFA with cyclophosphamide inhibited lung cancer growth and metastasis through a mechanism that involved decreased expression of CD4^+^CD25^+^Foxp3^+^ regulatory T cells (Tregs) and inhibition of the expression of transcription factors and cytokines associated with these cells. In addition, recent research has shown that the expression and function of CD4^+^CD25^+^Foxp3^+^Tregs and CD4^+^IL-17^+^ T helper 17 cells (Th17) are closely related. Therefore, we hypothesized that CD4^+^IL-17^+^ Th17 cells might be a key link between CD4^+^CD25^+^Foxp3^+^Tregs and tumor growth and metastasis. This implicates regulation of the balance between CD4^+^CD25^+^Foxp3^+^Treg and CD4^+^IL-17^+^ Th17 as a new strategy to reverse tumor progression.

The balance between CD4^+^CD25^+^Foxp3^+^Treg and CD4^+^IL-17^+^ Th17 cells plays an important role in the process of growth and metastasis. Both cell types are derived from the initial CD4^+^ T cell population and the differentiated cells perform opposing functions to maintain homeostasis for steady state immune function in the body [2, 3]. TGF-*β* and IL-6 are important factors in the initial differentiation of CD4^+^ T cells. TGF-*β* and IL-6 induce differentiation of CD4^+^ T cells into CD4^+^IL-17^+^ Th17 cells, which secrete IL-17 and IL-6 involved in inflammation and tumor development. TGF-*β* induces differentiation of CD4^+^ T cells into CD4^+^CD25^+^Foxp3^+^Tregs, which secrete TGF-*β* and express Foxp3 involved in immune regulation [4]. In addition, IL-23 and Th17 promote CD4^+^ T-cell differentiation, and IFN-*γ* inhibits CD4^+^ T-cell differentiation. In tumor microenvironment, overexpression of the above inflammatory cytokines disrupts the balance of CD4^+^CD25^+^Foxp3^+^Treg and CD4^+^IL-17^+^ Th17 [4], which can generate tumor growth signals and promote the spread of tumor cells to other tissues [5]. In the present study we examined the balance of these two CD4^+^ T-cell populations, the expression levels of proinflammatory cytokines, and the SOCS3-JAK2/STAT3 signal transduction pathway, to elucidate the mechanism underlying the effect of FZFA auxiliary treatment on the growth and metastasis of tumor.

## 2. Materials and Methods

### 2.1. Animals

This study was conducted using 6- to 8-week-old male C57BL/6 mice weighing 18–22 g. Animals were provided by The Experimental Animal Research Institute of the Chinese Academy of Medical Sciences (license number SCXK-11-00-0006). The Lewis lung cancer cell line was provided by Guanganmen Hospital of China Academy of Traditional Chinese Medicine (license number SYXK [Beijing] 2011-0001). Animals were housed under constant conditions of 23 ± 1°C and 40 ± 5% humidity and had free access to feed pellets and tap water. All animals were cared for in accordance with the policies and guidelines for animal use of the Chinese Academy of Traditional Chinese Medicine.

This study was approved by the Ethics Committee of the Chinese Academy of Traditional Chinese Medicine. All experiments were performed following the guidelines of the China Institute of Laboratory Animal Science (CALAS).

### 2.2. Preparation of FZFA

Tangshen (*Codonopsis* *pilosula*), Huangqi (*Astragalus* *mongholicus*), Gouqizi (medlar), Heshouwu (*Polygonum* *multiflorum*), Quanshen (bistort root), and Tengligen (Chinese* Actinidia* root) were purchased from Beijing Tongrentang Co. Ltd. The decoction of FZFA was composed of Tangshan (15 g), Huangqi (30 g), Gouqizi (12 g), Heshouwu (12 g), Quanshen (10 g), and Tengligen (12 g). FZFA crude powder was extracted twice using a 10-fold excess of boiling water and filtration through four-layer gauze. The combined filtrate was centrifuged at 2,000 rpm/min for 20 min and concentrated under reduced pressure. The concentrated solution was cooled to room temperature and mixed with pure alcohol to an alcohol content of 70%. The mixture was refrigerated for 24 h and filtered. The resulting sediment was vacuum-evaporated to obtain powder, which was dissolved in water immediately before oral administration. The FZFA decoction was concentrated to 1.3 g drug per milliliter. On the basis of the clinical dose for a 70 kg adult, we converted the dose for mice to 6.5 g crude drug per 100 g body weight.

### 2.3. Induction of Lewis Lung Cancer in Mice

The left armpit skin of the mice was disinfected with alcohol and 0.2 mL of a suspension of Lewis lung cancer cells (containing approximately 1 × 10^6^ cells/mL by trypan blue staining and counting of living cells) was injected into subcutaneous tissue using a 1 mL syringe.

### 2.4. Experimental Scheme

#### 2.4.1. Experimental Groups

Thirty-two C57BL/6 mice were randomly assigned to four groups as follows: FZFA + CTX treatment, CTX control, model control, and normal control (eight rats per group). Mice in the FZFA+CTX group received FZFA (6.5 g crude drug/100 g body weight for 3 weeks) plus cyclophosphamide (6 mg/100 g body weight), given by intraperitoneal injection within 48 h after inoculation. Mice in the CTX group received CTX (6 mg/100 g, given by intraperitoneal injection within 48 h after inoculation), while mice in the normal control groups received equivalent volumes of distilled water for 3 weeks without tumor cell injection and without drug.

#### 2.4.2. Assessment of Initial Immune Status

Blood samples (0.2 mL) were extracted from the inner canthus and mixed with heparin to prevent clotting. 20 *μ*L of monoclonal antibody reagents (Miltenyi Biotec, Germany) specific for CD3^+^, CD4^+^, CD8^+^, and NK cells was mixed to 180 *μ*L of blood and placed in the dark for 15 minutes. Blood cells were lysed using the Q-PREP system (Beckman-Coulter, Pasadena, CA). For each sample, the percentage of CD3^+^, CD4^+^, CD8^+^, and NK cells and the ratio of CD4^+^/CD8^+^ cells among 5,000 T cells were confirmed by flow cytometry (FACS CantoII, BD Biosciences). There was no significant difference in T-cell subsets between experimental groups before treatment (*P* > 0.05).

#### 2.4.3. Observation of Inhibition Rate of Lung Metastasis

21 days after inoculation, the mice were sacrificed under deep anesthesia. The lungs were rinsed with physiological saline, fixed with 4% paraformaldehyde for 24 h, dehydrated in various concentrations of ethanol, and embedded in paraffin. Sections of the pulmonary lobes (3 *μ*m thickness) were stained with hematoxylin and eosin. The number of metastases in every pulmonary lobe was counted (metastasis present as round small nodular protrusions with a circular translucent point of 2~5 mm; parts of metastases can be fused together). The inhibition rate of lung metastasis was evaluated as follows. Lung metastasis rate (%) = number of mice with metastasis/total number of mice. Inhibition rate of lung metastasis (%) = (1 − mean number of lung metastases in experimental group/mean number of lung metastases in control group) × 100.

#### 2.4.4. Tumor Volume and Weight

The tumors were stripped and then the mice and the tumors were weighed. Tumor volume (*V*) was calculated as *V* = *πabc*/2 (*a*: height, *b*: length, and *c*: width) [6].

#### 2.4.5. Determination of the Percentage and Ratio of Splenic CD4^+^IL-17^+^Th17 and  CD4^+^CD25^+^Foxp3^+^Treg Cells by Flow Cytometry Splenic CD4^+^CD25^+^Foxp3^+^Tregs

The spleens were removed and ground up, and spleen cells were separated from membrane and adipose tissue by filtration through a 300 mesh strainer. Spleen cells were resuspended in 2 mL PBS in a centrifuge tube and the number of cells was counted. Spleen cell suspension containing 1 × 10^6^ cells was added to l00 *μ*L PBS in a test tube, and 10 *μ*L PerCp anti-mouse CD3mAb (Miltenyi Biotec, Germany), 2 *μ*L FITC anti-mouse CD4mAb (Miltenyi Biotec), and 4 *μ*L PE anti-mouse CD25mAb (Miltenyi Biotec) were added. After incubation at 4°C in the dark for 30 min, the cells were centrifuged at 1500 rpm/min for 8 min, the supernatant was discarded, and the cells were resuspended in 1 mL of freshly prepared rupture of membrane liquid (Invitrogen, American) at 4°C for 40 min. After two washes with 2 mL 1x permeabilization buffer (Invitrogen, American), 4 *μ*L PEcy5 anti-mouse Foxp3 mAb (Miltenyi Biotec) was added and the mixture was incubated at 4°C in the dark for 30 min. For flow cytometric analysis, data were collected from 5,000 cells for each sample. With delineation of the lymphocyte group as the R1 gate and CD4^+^cell clusters as the R2 gate, Cell Quest software (BD Bioscience) was used to determine the percentage of CD4^+^CD25^+^Foxp3^+^Treg cells in the CD3^+^CD4^+^ T cells.

*Sp* *le* *ni* *c* *CD*4^+^ *IL*-17^+^ *Th*17. Spleen cells were isolated as above and incubated with 10 *μ*L PerCp anti-mouse  CD3 mAb (Miltenyi Biotec) and 2 *μ*L FITC anti-mouse CD4mAb (Miltenyi Biotec) at 4°C in the dark for 30 min. Cells were centrifuged at 1500 rpm/min for 8 min, the supernatant was discarded, and the cells were resuspended in 1 mL freshly prepared rupture of membrane liquid (Invitrogen, American) at 4°C for 40 min. After two washes with 2 mL 1x permeabilization buffer, 4 *μ*L PE anti-mouse IL-17 mAb (Miltenyi Biotec) was added and the mixture was incubated at 4°C in the dark for 30 min. Th17 cells were detected with flow cytometry (FACS CantoII, BD).

#### 2.4.6. Determination of the Percentage and Ratio of CD4^+^IL-17^+^Th17 and CD4^+^CD25^+^Foxp3^+^Treg Cells in Metastasis Foci by Immunohistochemistry

Lung tissue samples preserved in 4% paraformaldehyde solution were dehydrated and embedded in paraffin. The sections were deparaffinized and blocked with 3% peroxide-methanol at room temperature to remove endogenous peroxidase. Sections were incubated with 75 *μ*L of rabbit anti-human polyclonal antibody specific for IL-17 or Foxp3^+^ (Miltenyi Biotec) diluted in blocking buffer in a humidified chamber for 12 h, followed by incubation with 75 *μ*L of secondary antibody (Miltenyi Biotec) for 1 h in a humidified chamber. Subsequent immunostaining was performed using the biotin-streptavidin-peroxidase method with diaminobenzidine as a staining material and counterstaining with methyl green (1 min) or hematoxylin. Sections were immediately dehydrated in 70% ethanol, 80% ethanol, and 100% ethanol, and the glass slides were sealed with neutral gum. The negative control was treated as described above but the primary antibody was replaced by PBS. Sections were analyzed using a computerized image analyzer (ImageProPlus6, Media Cybernetics, American). The presence of brown granules in the cytoplasm or membrane of cells was classified as positivity; cells with no brown granules were classified as negative. Ten views were obtained for each slide, and seven slides were prepared for each organ. The percentage of positive cells was calculated as follows: (positive cells/total cells) × 100.

#### 2.4.7. Determination of IL-6, TGF-*β*, IL-17, IL-23, and IFN-*γ* Content by Enzyme-Linked Immunosorbent Assay (ELISA)

Culture supernatants of CD4^+^ T lymphocytes were collected into a 96-well plate. Test plates containing a blank well, 50 *μ*L of sample diluent, and standard samples were incubated at room temperature for 2 h, and then 100 *μ*L of a working solution of enzyme conjugates (Angiotensin, American) was added, and the plates were incubated at 37°C for a further 2 h. Serum concentrations of IL-17, IL-23, and IFN-*γ* were determined using the enzyme mark instrument (MullikanMK3, American). Absorbance (*A*) value was measured with a 450–630 nm dual wavelength. A standard curve constructed using the *A* values for the standard concentrations was used to determine the concentration of each cytokine in each sample.

#### 2.4.8. Quantitation of Splenic Foxp3 and *RORγt* mRNA by RT-PCR

Total RNA was extracted from the tissues using Trizol (Sangon, Shanghai, China) and its purity was determined using a Smart Spec TM3000 spectrophotometer (Bio-Rad, Hercules, CA, USA). cDNA was synthesized according to the manufacturer's instructions (Genechem, Shanghai). PCR was performed using a GeneAmp 9600 (Perkin Elmer, Waltham, MA, USA) with cDNA as template and TaqDNA, 10x buffer, MgCl2, dNTPs, and forward and reverse primers for GAPDH, Foxp3, and ROR*γ* (Sangon Shanghai, China). The primer sequences used were Foxp3, forward primer 5′-CTGACCAAGGCTTCATCTGT-3′ and reverse primer 5′-AACTCTGGGAATGTGCTGTT-3′; ROR*γ*, forward primer 5′-GGCTCCCTGGATGAATAGAATG-3′ and reverse primer 5′-AGGCGAGGCAGAAAATGTAAAG-3; GAPDH, forward primer 5′-CCTTCATTGACCTCAACTACATG-3′ and reverse primer 5′-CTTCTCCATGGTGGTGAAGAC-3′. The PCR reaction conditions were 95°C for 5 min, 40 cycles of 95°C for 25 s, 55°C for 25 s, 72°C for 50 s, and final extension at 72°C for 5 min. The reaction products were subjected to 2% agarose gel electrophoresis at 60 V for 2 h and observed under an ultraviolet lamp. Expression of Foxp3 and ROR**γ**t mRNA was determined semiquantitatively using Fluor-S MultiImager (Bio-Rad), with *GAPDH* as an internal reference. The gray scale (or opacity degree, OD) of each band was screened and the densitometric ratio for Foxp3 and ROR**γ** relative to *GAPDH* was calculated for each sample [7, 8].

#### 2.4.9. Determination of Splenic JAK2, STAT3, and SOCS3 Protein by Western Blotting

Spleen tissue (100 mg) was cut into small pieces and mixed with lysate, and the suspension was homogenized and centrifuged. Protein concentration was determined by the Bradford method, and tissue was preserved at −80°C prior to testing. A 50 *μ*g sample of tissue from each group was used for 15% SDS-PAGE. Proteins were transferred to a PVDF membrane, which was blocked in 5% nonfat milk powder for 1 h at room temperature before incubation with antibodies specific for JAK2, STAT3, and SOCS3 (Cell Signaling Technology Boston, MA, USA; diluted 1 : 2,000 in TBST buffer) at 4°C overnight. Membranes were washed twice in TBST (10 min each wash) and then visualized with electrochemiluminescence. Image analysis was performed with Image Master VDS software (Amersham Pharmacia Biotech, Uppsala, Sweden) using GAPDH as an internal control to determine the relative protein concentration.

### 2.5. Statistical Analysis

All data were expressed as means ± standard errors of the mean (SEMs). Number of experiments (*n*) is indicated in the legend of each figure. All analysis was performed using the statistical package for the social sciences (SPSS) statistical software for Windows, version 17.0 (SPSS Inc., USA). The statistical significance of differences was assessed by one-way ANOVA. *P* < 0.05 was considered to be significantly different. When ANOVA indicated a significant difference, LSD or Dunnett's T3 posthoc test was then used to assess the difference between groups.

## 3. Results

### 3.1. Tumor-Burden Mouse, Armpit Implantation Tumor, and Lungs of Tumor-Burden Mice in Each Group

See Figure 1.

### 3.2. Pathologic Analysis of Each Group

**Figure 2:**
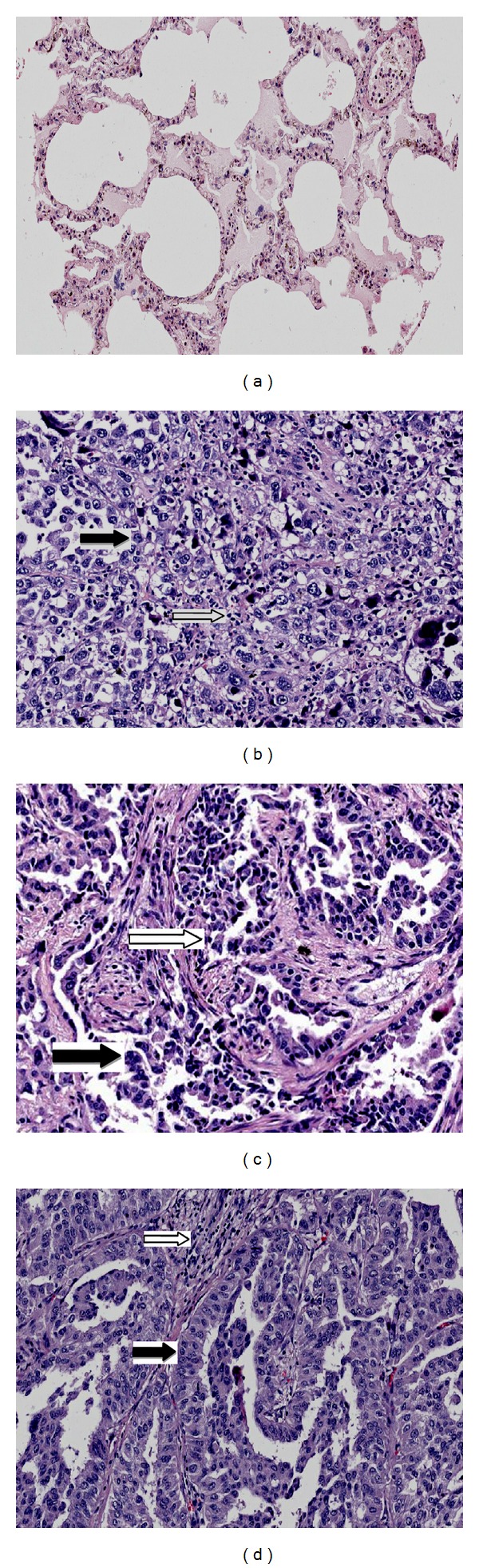
The pathological picture of normal control group (a), model control group (b), CTX group (c), and FZFA + CTX group (d). Lung tissues were fixed, sectioned at 4 *μ*m thickness, stained with H&E solution, and observed under a microscope of 200 magnifications. White arrows indicate inflammatory cells and black arrows indicate the cancer cells.

The lungs of mice in the normal control group had alveolar walls with structural integrity and no collapse; there was no alveolar secretion or infiltration of inflammatory cells (Figure 2(a)). Lungs of mice in the model control group had a large number of metastatic cancer cells exhibiting nuclear hyperchromatism and irregular shape; the alveolar walls were thickened and there were large numbers of infiltrative mononuclear cells (Figure 2(b)). In comparison, the range of mononuclear cell infiltration in the lungs of mice in the CTX group (Figure 2(c)) and FZFA+CTX group (Figure 2(d)) was smaller, and the alveolar structure was relatively complete. The pathology of lungs in mice in the FZFA + CTX group was less disrupted than that of the CTX group.

### 3.3. Inhibition Rate of Lung Metastasis of Each Group

The Number of lung metastases in the model control group was higher than that in the other groups. Number of lung metastases in the FZFA + CTX group was significantly lower than that of the CTX group (*P* < 0.05). The inhibition rate of lung metastasis in the model control group was 0. The inhibition rate of metastasis in the FZFA + CTX group was higher than that of the CTX group (Table 1).

### 3.4. Tumor Volume and Weight of Each Group

The volume and weight of tumors in the model control group were higher than those in the other groups. Tumor weight and volume in the FZFA + CTX group were significantly lower than those of the CTX group (*P* < 0.05) (Table 2).

### 3.5. Percentage and Ratio of Splenic CD4^+^IL-17^+^Th17 and CD4^+^CD25^+^Foxp3^+^Treg Cells

**Figure 3:**
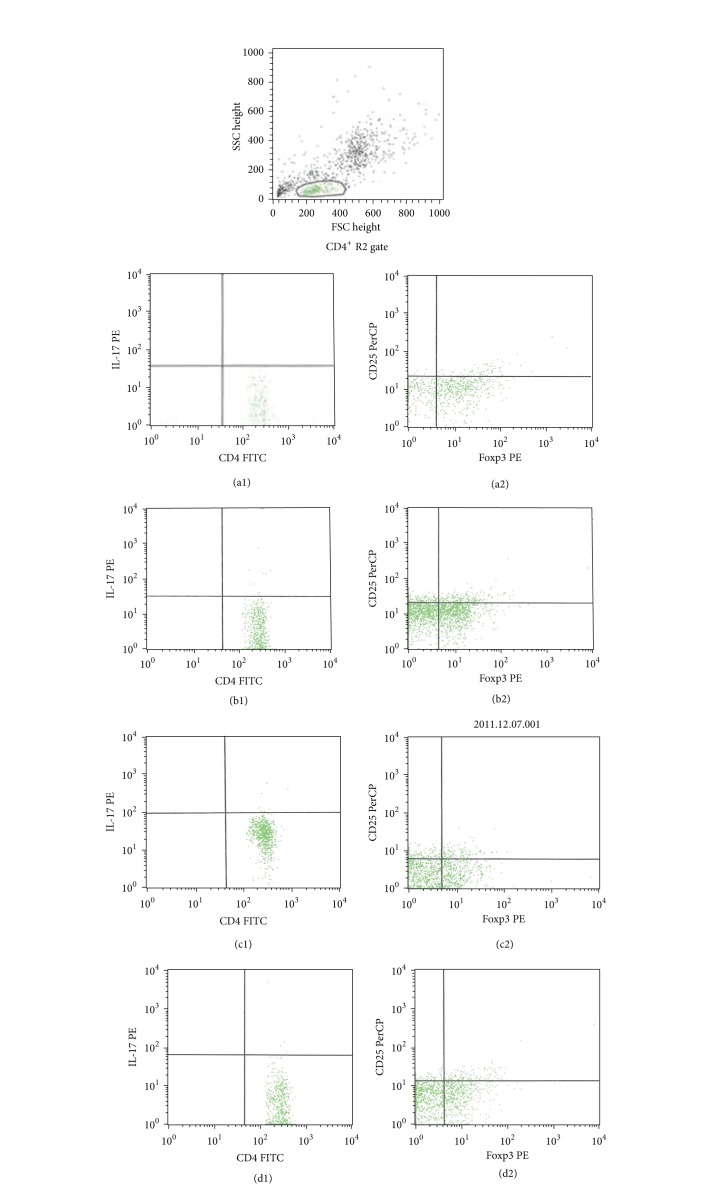
The distribution plot of Thl7 and Treg. Normal control group Th17 (a1), normal control group Treg (a2); model control group Th17 (b1), model control group Treg (b2); CTX group Th17 (c1), CTX group Treg (c2), FZFA+CTX group Th17 (d1), and FZFA + CTX group Treg (d2). Spleen cells were separated and labeled by antibodies and then detected by flow cytometry.

The percentage of CD4^+^IL-17^+^  Th17 and CD4^+^CD25^+^Foxp3^+^Tregs in the spleen was very low in the normal control group but significantly higher in the model control group (*P* < 0.05). The percentage of splenic CD4^+^IL-17^+^  Th17 and CD4^+^CD25^+^Foxp3^+^Tregs in the FZFA+CTX group was significantly lower than that of the CTX group (*P* < 0.05; Table 3, Figure 3).

More importantly, the ratio of Thl7/Treg approached normalization only in the in the FZFA + CTX group; the ratio of Thl7/Treg in the FZFA + CTX group was not significantly different from that of the normal group (*P* < 0.05; Table 3).

### 3.6. CD4^+^IL-17^+^Th17 and CD4^+^CD25^+^Foxp3^+^ **Treg** in Metastasis Foci (Table 4, Figure 4)

**Figure 4:**
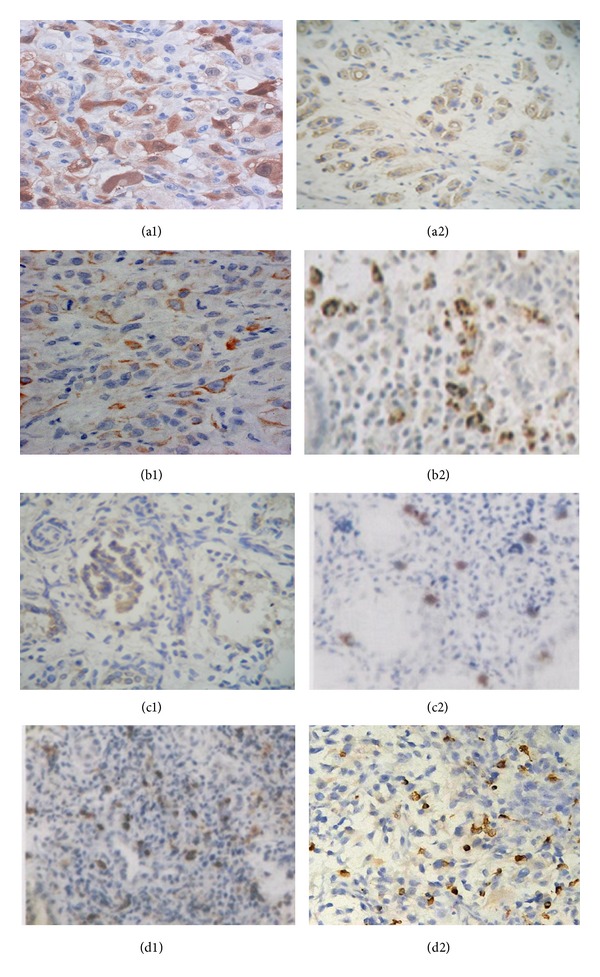
The Th17 and Treg of metastasis foci detected by immunohistochemistry. Normal control group Th17 (a1), normal control group Treg (a2); model control group Th17 (b1), model control group Treg (b2); CTX group Th17 (c1), CTX group Treg (c2), FZFA + CTX group Th17 (d1), and FZFA + CTX group Treg (d2). Lung tissues were fixed, sectioned at 4 *μ*m thickness, and incubated with primary antibody and secondary antibody; then the percentage of positive cells was calculated under a microscope of 400 magnifications.

The percentage of CD4^+^IL-17^+^ Th17 and CD4^+^CD25^+^Foxp3^+^Treg cells in the metastatic foci was very low in the normal control group but was significantly increased in the model control group (*P* < 0.05). The percentage of splenic CD4^+^IL-17^+^ Th17 and CD4^+^CD25^+^Foxp3^+^ Treg in the FZFA + CTX group was significantly lower than in the CTX group (*P* < 0.05) (Table 4, Figure 4).

More importantly, the ratio of Thl7/Treg approached normalization only in the FZFA + CTX group. The ratio of Thl7/Treg in the FZFA + CTX group showed no significant difference from that of the normal group (*P* < 0.05; Table 4).

### 3.7. Content of IL-6, TGF-*β*, IL-17, IL-23, and IFN-*γ*

The model control group showed a significant increase in serum levels of IL-17, IL-23, and IFN-*γ* compared with the normal control group (*P* < 0.01). Expression in the FZFA + CTX group and CTX group was significantly lower than that in the model control group (*P* < 0.05). Moreover, there was a significant difference between expression in the FZFA+CTX group and the CTX group (*P* < 0.05; Table 5).

### 3.8. Quantitation of Splenic Foxp3 and ROR**γ**t mRNA Expression

**Figure 5:**
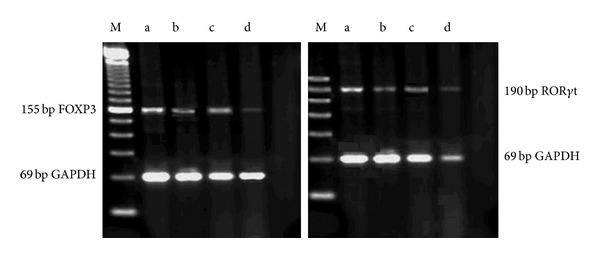
The Foxp3 and ROR**γ**t mRNA electrophoresis by RT-PCR. Model control group (a), normal control group (b), CTX group (c), FZFA + CTX group (d), and M: marker. GAPDH as internal reference.

The mRNA expression of Foxp3 and ROR**γ**t in the spleen was very low in the normal control group but significantly higher in the model control group (*P* < 0.05). In contrast, the expression of Foxp3 and ROR**γ**t mRNA in the FZFA + CTX group was significantly lower than that in the CTX group (*P* < 0.05; Table 6, Figure 5).

### 3.9. Protein Expression of Splenic JAK2, STAT3, and SOCS3

**Figure 6:**
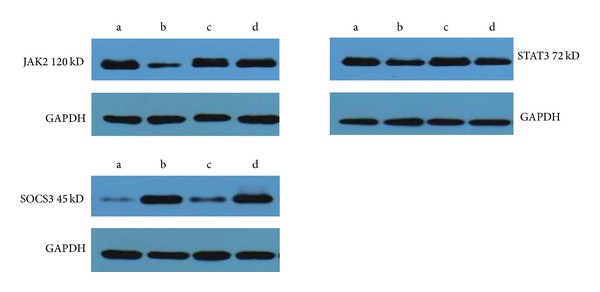
The JAK2, STAT3, and SOCS3 protein electrophoresis by Western blotting. Model control group (a), normal control group (b), CTX group (c), and FZFA + CTX group (d). GAPDH as internal reference.

SOCS3 protein expression was significantly downregulated in all groups except for the normal control group (*P* < 0.05). JAK2 and STAT3 protein expression was significantly higher in the model control group than in all other groups (*P* < 0.05). The protein expression of SOCS3, JAK2, and STAT3 in the FZFA + CTX group was lower than that in the CTX group (*P* < 0.05; Table 7, Figure 6).

## 4. Discussion

Our previous studies showed that FZFA+CTX had greater anticancer efficacy than CTX alone, including greater inhibition of mouse cancers, longer life span, and improved survival curves [9–11]. Cytologic studies showed that FZFA + CTX had obvious inhibitory effects on cancer cells, decreasing the mitotic index and DNA content and shifting the DNA histogram to the left [9, 12].

The CD4^+^CD25^+^Foxp3^+^ Treg cells that mediate immune tolerance and the CD4^+^IL-17^+^ Th17 cells that mediate the inflammatory response are derived from the initial T lymphocyte cells. Their functions and differentiation processes are opposing and under normal circumstances keeping these two cell populations in balance helps maintain immune stability [5, 13]. However, during tumor formation and development the balance of CD4^+^IL-17^+^ Th17 and CD4^+^CD25^+^Foxp3^+^Treg can be disrupted by inflammatory cytokines [14]. Exploring the interaction between CD4^+^CD25^+^Foxp3^+^Treg and CD4^+^IL-17^+^ Th17 cells has great implications for understanding regulation of the immune inflammatory microenvironment and for the clinical application of traditional Chinese medicine in the reversion of tumor growth and tumor immune escape.

In this study, the FZFA+CTX group exhibited less tumor growth and a lower metastasis rate of Lewis lung cancer than the other groups. The expression of CD4^+^IL-17^+^ Th17 and CD4^+^CD25^+^Foxp3^+^Treg in each group showed a rising trend in all groups, but expression in the FZFA + CTX group was significantly lower than that in the other groups (*P* < 0.05). Moreover, the ratio of CD4^+^IL-17^+^ Th17 to CD4^+^CD25^+^Foxp3^+^Treg was unbalanced in all groups except for the FZFA + CTX group. Overall, the percentage of CD4^+^IL-17^+^ Th17 and CD4^+^CD25^+^Foxp3^+^Treg was concordant with the extent of disease.

ROR*γ*t and Foxp3 are specific nuclear transcription factors for CD4^+^IL-17^+^ Th17 and CD4^+^CD25^+^Foxp3^+^Treg, respectively [15]. SOCS3-JAK2/STAT3 is the main signal transduction pathway mediating the differentiation of CD4^+^CD25^+^Foxp3^+^Treg and CD4^+^IL-17^+^ Th17 [16]. The expression of IL-6, TGF-*β*, IL-17, and IL-23 was directly proportional to the levels of CD4^+^IL-17^+^ Th17 and CD4^+^CD25^+^Foxp3^+^Treg, whereas expression of IFN-*γ* showed an inverse relationship. Moreover, changes in expression of the transcription factors except for SOCS3, the Foxp3, ROR**γ**t, and the signaling molecules JAK2 and STAT3 also corresponded with changes in CD4^+^IL-17^+^ Th17 and CD4^+^CD25^+^Foxp3^+^Treg expression.

## 5. Conclusion

Traditional Chinese medicine combined with chemotherapy inhibited the tumor growth, metastasis, and the immune inflammatory response more efficiently than chemotherapy alone. Therefore, FZFA+CTX and CTX alone had inhibitory effects on the tumor growth and metastasis, but FZFA+CTX had greater activity than CTX alone.

## Figures and Tables

**Figure 1:**
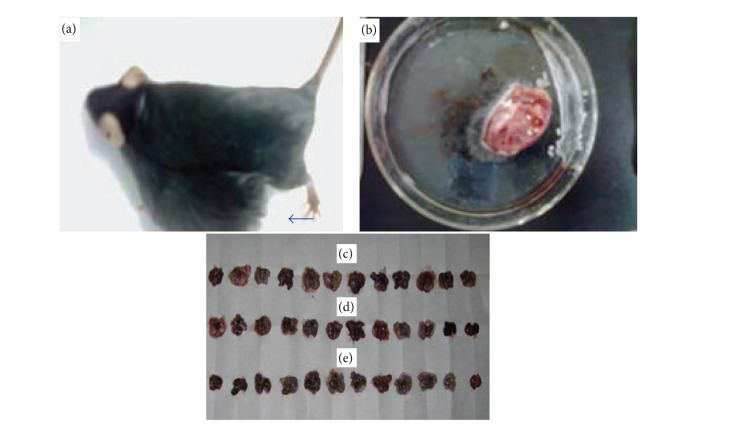
Judging from the appearance of armpit implantation tumor (b) in tumor-burden mouse (a), the size of armpit implantation tumors in descending order is the model control group, the CTX group, and the FZFA + CTX group. Seen with the naked eye, the lungs of tumor-burden mice were not significantly different between the model control group (c), the CTX group (d), and the FZFA + CTX group (e).

**Table 1:** Inhibition rate of lung metastasis (mean ± SE).

Group	*n*	Metastasis rate (%)	Number of lung metastases (*n*)	Inhibition rate of lung metastasis (%)
Normal group	8	*∖*	*∖*	*∖*
Model group	8	100	13.50 ± 0.91^△^	0
CTX group	8	75	10.50 ± 1.25^∗△^	26.65
FZFA + CTX group	8	62.5	7.63 ± 0.63*	50.94

*P < 0.05, versus model control group; ^△^ *P* < 0.05, versus FZFA + CTX group.

**Table 2:** Volume and weight of tumor (mean ± SE).

Group	*n*	Body weight (g)	Tumor weight (g)	Tumor volume (cm^3^)
Normal group	8	21.9 ± 1.58	0	0
Model group	8	22.8 ± 1.44	4.53 ± 1.03^△^	3.06 ± 0.84^△^
CTX group	8	20.4 ± 0.95	3.43 ± 0.93^∗△^	2.04 + 0.55^∗△^
FZFA + CTX group	8	22.0 ± 2.01	2.12 ± 0.79*	1.38 + 0.32*

*P < 0.05, versus model control group; ^△^ *P* < 0.05, versus FZFA + CTX group.

**Table 3:** Percentage and ratio of splenic Th17 and Treg (mean ± SE).

Group	*n*	Thl7 (%)	Treg (%)	Th17/Treg
Normal group	8	0.64 ± 0.01^∗△^	5.52 ± 0.10^∗△^	0.114 ± 0.004
Model group	8	1.20 ± 0.04^△^	8.96 ± 0.91^△^	0.132 ± 0.012^*※*^
CTX group	8	1.04 ± 0.06^∗△^	8.25 ± 0.34^∗△^	0.127 ± 0.012^*※*^
FZFA + CTX group	8	0.78 ± 0.11*	7.03 ± 0.14*	0.111 ± 0.015

*P < 0.05, versus model control group; ^△^ *P* < 0.05, versus FZFA + CTX group. ^*※*^ *P* < 0.05, versus the normal control group.

**Table 4:** Percentage and ratio of Th17 and Treg of metastasis foci (mean ± SE).

Group	*n*	Thl7 (%)	Treg (%)	Th17/Treg
Normal group	6	11.40 ± 1.15*	2.52 ± 0.40*	4.57 ± 1.09
Model group	6	34.69 ± 3.85^△^	11.50 ± 0.44^△^	3.02 ± 0.39^*※*^
CTX group	6	18.87 ± 1.78^∗△^	5.91 ± 0.27^∗△^	3.17 ± 0.49^*※*^
FZFA + CTX group	6	11.40 ± 0.08*	2.75 ± 0.30*	4.17 ± 0.69

*P < 0.05, versus model control group; ^△^ *P* < 0.05, versus FZFA + CTX group. ^*※*^ *P* < 0.05, versus the normal control group.

**Table 5:** Content of IL-6, TGF*β*, IL-17, IL-23, and IFN-*γ* (mean ± SE).

Group	*n*	IL-6 (pg/mL)	TGF*β* (pg/mL)	IL-17 (pg/mL)	IL-23 (pg/mL)	IFN*γ* (pg/mL)
Normal group	8	0.11 ± 0.04*	0.08 ± 0.004*	0.14 ± 0.02*	0.38 ± 0.05*	0.96 ± 0.29*
Model group	8	0.20 ± 0.04^△^	0.17 ± 0.03^△^	0.18 ± 0.07^△^	0.63 ± 0.14^△^	2.72 ± 0.62^△^
CTX group	8	0.18 ± 0.03^∗△^	0.16 ± 0.01^△^	0.17 ± 0.02	0.40 ± 0.15*	1.94 ± 0.40^∗△^
FZFA + CTX group	8	0.11 ± 0.002*	0.09 ± 0.01*	0.15 ± 0.03*	0.39 ± 0.02*	1.15 ± 0.54*

*P < 0.05, versus model control group; ^△^ *P* < 0.05, versus FZFA + CTX group.

**Table 6:** Quantitation of splenic Foxp3 and ROR*γ*t mRNA (Mean ± SE).

Group	*n*	Foxp3 mRNA	ROR*γ*t mRNA
Model group	8	1.81 ± 0.08^△^	1.91 ± 0.14^△^
Normal group	8	1.10 ± 0.10*	1.19 ± 0.06*
CTX group	8	1.53 ± 0.05^∗△^	1.58 ± 0.07^∗△^
FZFA + CTX group	8	1.12 ± 0.03*	1.19 ± 0.80*

*P < 0.05, versus model control group; ^△^ *P* < 0.05, versus FZFA + CTX group.

**Table 7:** Protein expression of splenic JAK2, STAT3, and SOCS3 (mean ± SE).

Group	*n*	JAK2	STAT3	SOCS3
Model group	8	28.96 ± 3.71^△^	21.88 ± 1.11^△^	3.38 ± 0.13^△^
Normal group	8	12.18 ± 1.00^∗△^	17.40 ± 2.10*	10.01 ± 1.31*
CTX group	8	23.71 ± 5.01^∗△^	19.82 ± 2.09^∗△^	7.43 ± 0.11^∗△^
FZFA + CTX group	8	19.12 ± 0.40*	17.78 ± 1.12*	10.00 ± 1.15*

*P < 0.05, versus model control group; ^△^ *P* < 0.05, versus FZFA + CTX group.

## References

[1] Coussens LM, Werb Z (2002). Inflammation and cancer. *Nature*.

[2] Liu LM, Zhen C, Chen PF (2007). The traditional Chinese medicine in the treatment of malignant tumor. *Journal of Traditional Chinese Medicine*.

[3] Yu MW, Sun GZ, Li DR (2009). The role of Hematoxylin, SuMu Astragalus on Lewis lung cancer bearing mice spleen dendritic cells of traditional Chinese medicine. *Chinese Journal of Integrative Medicine*.

[4] Chen XM (2008). Effect of inflammation on the biological behavior of malignant tumor. *Basic and Clinical Tumor*.

[5] Wang WW, Shen Q (2009). Th17 cells and its relationship with development of tumors. *Tumor*.

[6] He ZJ, Chen XX (2009). Comparison of different calculation methods of tumor volume. *Chinese Journal of Comparative Medicine*.

[7] Chen G (2007). Liver lipid molecules induce PEPCK-C gene transcription and attenuate insulin action. *Biochemical and Biophysical Research Communications*.

[8] De WL, Lim HW, Liang Q (2001). Targeted inhibition of calcineurin attenuates cardiac hypertrophy in vivo. *Proceedings of the National Academy of Sciences*.

[9] Sun GZ, Wang GM, Chen CH (1995). Fuzhengfangai liquid combined with chemotherapy in treatment of advanced gastrointestinal cancer clinical and experimental research. *Journal of Surgery Combined with Traditional Chinese Medicine and Western Medicine Chinese*.

[10] Zhang PX, Yu GQ, Pu BK (1996). The regulation of Feiliuping and righting anti-cancer plaster on serum ascorbate free radical in mice. *Journal of Surgery Combined with Traditional Chinese Medicine and Western Medicine China*.

[11] Xue YP (2013). Clinical and experimental studies of adjuvant treatment for colon cancer liver metastasis fuzhengfangai liquid Chinese folk therapy. *China's Naturopathy*.

[12] Zhang PT, Sun GZ, Duan SJ, Gao M, Yang Z (1994). Scavenging effects of Fuzheng anticancer ointment on the enzymatic and non enzymatic system of hydroxyl free radical and superoxide anion. *Chinese Journal of Pharmaceuticals*.

[13] Infante-Duarte C, Horton HF, Byrne MC, Kamradt T (2000). Microbial lipopeptides induce the production of IL-17 in Th cells. *Journal of Immunology*.

[14] Chen XM (2008). Effect of inflammation on the biological behavior of malignant tumor. *Basic and Clinical Tumor*.

[15] Ivanov II, McKenzie BS, Zhou L (2006). The orphan nuclear receptor ROR*γ*t directs the differentiation program of pro-inflammatory IL-17^+^ T helper cells. *Cell*.

[16] Yu JH, Kim KH, Kim H (2008). SOCS 3 and PPAR-*γ* ligands inhibit the expression of IL-6 and TGF-*β*1 by regulating JAK2/STAT3 signaling in pancreas. *International Journal of Biochemistry and Cell Biology*.

